# Chronic inflammatory demyelinating polyneuropathy and psoriasis comorbidity with significantly alleviated in symptoms after secukinumab: case report

**DOI:** 10.1186/s12883-022-02928-3

**Published:** 2022-11-02

**Authors:** Yan Jin, Hong Chu, Hongjuan Dong, Hongyang Wang, Qingping Wang, Xiaoquan Zhao, Dongdong Qin, Zuneng Lu, Chao Weng

**Affiliations:** Department of Neurology, Renmin Hospital of Wuhan University, Hubei Province, Wuhan, 430060 People’s Republic of China

**Keywords:** Chronic inflammatory demyelinating polyneuropathy, Psoriasis, Immunomodulatory therapy, Treatment, Case report

## Abstract

**Background:**

Chronic inflammatory demyelinating polyneuropathy (CIDP) is an autoimmune disease that involves damage to the peripheral nervous system. The course of the disease can progress for more than 8 weeks, with frequent incidences of relapse-remission courses. This article reported a rare combination of CIDP with fluctuating symptoms, recurrence-remission, and comorbidity with psoriasis.

**Case presentation:**

A 29-year-old male patient with repeated limb weakness and numbness was admitted to the hospital several times in the past six months. He had a history of psoriasis for 6 years, and the medications (clobetasol propionate ointment and calcipotriol ointment) treated for psoriasis were discontinued 1 year ago. During the hospitalization, repeated intravenous injections of human immunoglobulin G (IVIg), immunoadsorption, and secukinumab were performed. Nerve electrophysiology tests, ganglioside autoantibody spectrum tests, and clinical MRC muscle strength scores were performed on a regular basis to confirm the diagnosis of CIDP. The patient was regularly followed up.

**Results:**

After repeated rounds of human IVIg and immunoadsorption, the patient’s MRC score was increased by ≥ 6 points. The first ganglioside autoantibody spectrum test showed anti-GQ1b IgG ( +) and anti-GM1 IgM ( +) antibodies, and all were negative after re-examination. Finally, the patient was treated with the IL-17A inhibitor secukinumab for psoriasis. During 7 months of follow-up, the CIDP and psoriasis symptoms are relatively stable.

**Conclusion:**

Combination of IVIg and immunoadsorption was highly effective in treating CIDP complicated with psoriasis. The clinical manifestations of CIDP are diverse. When relapse-remission occurs in the course of the disease, it is necessary to clarify whether it is combined with other autoimmune diseases and should control the autoimmune diseases as soon as possible.

**Supplementary Information:**

The online version contains supplementary material available at 10.1186/s12883-022-02928-3.

## Background

Chronic inflammatory demyelinating polyneuropathy (CIDP) is characterized by the progressive loss of myelin sheaths on the peripheral nerve fibers, leading to impaired motor and sensory functions in the legs and arms and hyperactivation of neuroinflammatory responses as well [1]. As reports indicate, CIDP accounts for the overall prevalence rate of 0.67 ~ 10.3 cases per 100,000 persons, with an incidence rate of 0.15 ~ 10.6 cases per 100,000 individuals [2]. Although the pathomechanistic involvement of immune B and T cells has been implicated in the disease onset and progression, however, the exact underlying mechanisms remain unclear to date. Notably, CIDP exhibits diverse clinic-pathological symptoms, and the treatment outcomes vary greatly between the individual patients. Because of the significant contribution of the secondary complications, diagnostic precision and selection of the best possible therapeutic strategy have been challenging. Psoriasis is a common chronic skin disease associated with systemic inflammatory alterations in combination with the genetic and/or environmental etiology [3]. The prevalence of psoriasis is relatively less in the Chinese population (0.3%) [4] compared to the Europeans and North Americans (2%) [5]. Therefore, the concurrence of CIDP and psoriasis is a rare disease phenotype in any population. Here, we reported a rare case of CIDP combined with plaque psoriasis to further enrich our understanding of the diagnosis and treatment options and to reveal the underlying pathomechanisms.

## Case presentation

**Fig. 1:**
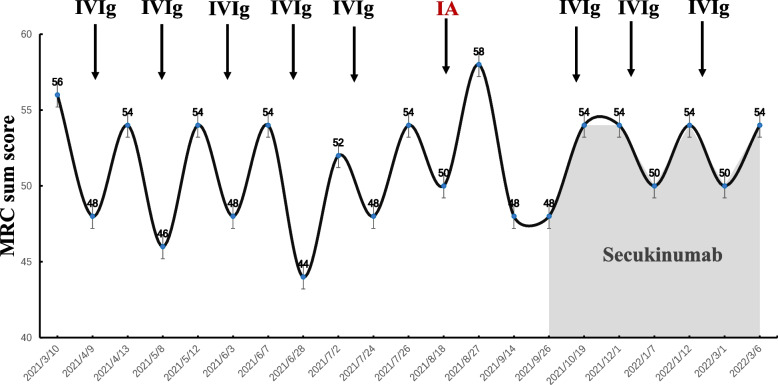
Schematic diagram of symptom relief-recurrence fluctuation after immunomodulatory treatment. After each IVIG treatment, the symptoms improved, and the MRC sum scores were increased by ≥ 6 points. The patient was treated with secukinumab on September 27^th^, 2021, at a dose of 300 mg subcutaneous injection (1 time/week, for 5 weeks). During the follow-up, the patient's clinical symptoms have been stabilized for a significantly longer period of time than before. (Abbreviations: IVIg: intravenous injections of human immunoglobulin G; IA: immunoadsorption)

A 29-year-old man was re-admitted to our hospital on September 9^th^, 2021, presenting with the symptoms of repeated limb weakness and numbness for half a year, and then suddenly aggravated for the past 3 days prior to the hospitalization. Six months ago, the patient reportedly had symptoms of muscle weaknesses in hands and legs, such as extreme difficulties in hand straightening, twisting bottle cap, standing up after squatting, and restricted walking ability as well, without any obvious cause. Later, he developed numbness in his limbs and muscle aches. During his initial visits to another hospital, he was initially diagnosed with the" Guillain-Barré syndrome ", which was later modified to be diagnosed with CIDP, and prescribed oral prednisone tablets (40 mg/day) and mycophenolate mofetil dispersible tablets (Seccopine) (0.75 g/morning and 0.5 g/night) in early April. However, these drugs could not significantly improve his symptoms, so he stopped the treatment in August. During this period, he underwent 5 courses of intravenous immunoglobulin (IVIg) therapy (0.4 g/kg/d, 5 days/course, 1 time/month) in another hospital. He subjective assumed that each IVIg therapy could significantly ameliorate the muscle weakness symptoms; and the Medical Research Council (MRC) sum scores were (0–60) increased by ≥ 6 points (Fig. 1). However, there was a recurrence of symptoms with further worsening in about half a month after each course. The patient was admitted to our department on August 4^th^, 2021 due to aggravated limb weakness. Then, he was subjected to 3 courses of immunoadsorption (IA) treatment, which happened to relieve the symptoms completely. The MRC sum scores were increased to 58 points. He was discharged from our hospital on August 27^th^, 2021 and received oral azathioprine (100 mg/day). During this period, patients received acupuncture and exercise therapy as rehabilitation training. Unfortunately, his limb weakness and numbness drastically increased on September 6^th^, 2021, which brought him to our hospital for further treatment. The patient’s past medical history indicated suffering from psoriasis for 6 years and treatment with clobetasol propionate ointment and calcipotriol ointment on an intermittent basis. He stopped taking the medications 1 year ago, resulting in the recurrence of more severe symptoms. Following that, the patient underwent traditional Chinese medicine therapy, including the mung bean bath as per the physician’s direction. Immediately after that, the patient experienced increased severity of muscle weakness and psoriasis plaques. Dermatological examinations revealed patchy hypopigmentation spots on the skin areas of his torso and limbs, slightly scaly texture on the upper limbs, and erythema on his external auditory canal and scalp. His limb muscle strength test showed grade 4 on the MRC Scale for muscle strength with normal limb muscle tone. Clinically, his left biceps reflex disappeared, other upper limb tendon reflexes were weakened, lower limb tendon reflexes disappeared. His superficial sensations were slightly decreased. The auxiliary tests, including cerebrospinal fluid (CSF) biochemistry and immunity, exhibited significant upregulation of total protein (1424 mg/L) and albumin (770 mg/L), in addition to IgG (169.0 mg/L) and IgA (38.5 mg/L). Diffusion-weighted magnetic resonance imaging (MRI) of the head with contrast enhancement could not detect any abnormalities. On May 8^th^, 2021the patient was tested for the ganglioside autoantibody panel titers indicating positivity for both anti-GQ1b IgG and IgM anti-GM1 antibodies. Further examination of the ganglioside autoantibody spectrum on September 10^th^, 2021, showed negative results. His previous (April 7^th^, 2021) nerve electrophysiology test results indicated multiple peripheral nerve damage foci in the extremities, primarily due to demyelination of motor and sensory fibers. The F wave latency of the extremities was significantly prolonged with the involvement of nerve roots. Two consecutive re-examination results on August 5^th^ and 16^th^ consistently showed multiple peripheral nerve damages, including both proximal and distal nerve roots, demyelination combined with axonal damage, among which demyelination was the most severe. Multiple nerve motor conduction blocks could be observed (Table 1). Therefore, this patient fulfilled the recent diagnostic criteria of the typical CIDP [1].

**Table 1:** Nerve conduction test results

	2021/04/07		2021/08/05		2021/08/16	
	Left	Right	Left	Right	Left	Right
**Motor conduction studies** **Median nerve**						
DML (ms)	5.58 *	7.4 *	8.71 *	9.04 *	8.84 *	10.5 *
CMAP (mV)	9.7	4.66 *	3 *	6.5	5.3 *	7.3
CV (elbow-wrist, m/s)	20.9 *	22.9 *	17.5 *	17.2 *	15.2 *	16.7 *
F—wave latency (ms)	33.3	30.4	-	-	NR	48.6 *
F-wave persistence(%)	40 *	43.8 *	-	-	NR	40 *
**Ulnar nerve**						
DML (ms)	3.66 *	4.5 *	5.42 *	5.17 *	5.15 *	5.32 *
CMAP (mV)	5.5 *	3.49 *	4.2 *	5.8 *	4.5 *	5.4 *
CV (below elbow-wrist, m/s)	29.1 *	23.2 *	23.2 *	16.7 *	19.8 *	21.3 *
F—wave latency (ms)	28.2	70.4 *	-	-	NR	NR
F-wave persistence(%)	40 *	60 *	-	-	NR	NR
**Tibial nerve**						
DML (ms)	4.95	7.6 *	6.77 *	5.92 *	6.74 *	6.25 *
CMAP (mV)	19.8	18.08	0.4 *	4.9 *	4.6 *	5.4 *
F—wave latency (ms)	93.6 *	96.8 *	-	-	NR	NR
F-wave persistence(%)	100	68.8 *	-	-	NR	NR
**Peroneal nerve**						
DML (ms)	5.98 *	7.6 *	8.5 *	8.54 *	8.42 *	10.4 *
CMAP (mV)	5.8	6.48	3.3	3.2	3.9	4.4
CV (fibular neck-ankle, m/s)	34.1 *	30.6 *	23.5 *	24.2 *	25.8 *	26.7 *
F—wave latency (ms)	89.5 *	-	-	-	NR	NR
F-wave persistence(%)	45.5 *	-	-	-	NR	NR
**Sensory conduction studies**						
**Median nerve**						
Peak latency (ms)	3.53	4.2	4.98	4.34	5.61	5.96
The SNAP (μV)	10.3 *	7.73 *	5.5 *	7.5 *	8.3 *	4.4 *
CV (wrist-index finger, m/s)	34.8	32.7 *	28.1 *	32.3 *	25 *	23.5 *
**Ulnar nerve**						
Peak latency (ms)	3.74	3.9	4.09	3.86	4.77	4.77
The SNAP (μV)	12.7 *	11.52 *	11.1 *	8.2 *	10.6 *	12.5 *
CV (wrist-little finger, m/s)	29.9 *	29.7 *	28.1 *	28.5 *	24.1 *	23.1 *
**Radial nerve**						
Peak latency (ms)	5.15	-	3.9	3.64	3.76	3.86
The SNAP (μV)	5.2 *	-	8.4 *	9.6 *	4.5 *	7.2 *
CV (forearm-snuff box, m/s)	36.5 *	-	30.8 *	31.6 *	34.6 *	29.8 *
**Sural nerve**						
Peak latency (ms)	2.63	2.9	2.22	2.02	2.16	2.15
The SNAP (μV)	17.5	11.51	11.8	14.7	19.1	27.8
CV (calf-ankle, m/s)	45.6	44.2	47.3	52	50.9	53.5
**Superficial peroneal nerve**						
Peak latency (ms)	-	3.2	2.41	2.6	2.47	2.69
The SNAP (μV)	-	21.35	20.5	13.9	20.9	19
CV (lower leg-ankle, m/s)	-	40.6	45.6	44.2	44.5	42.8

*NR*  no response, Absence of test, *Abnormal values

Abbreviations: *DML* distal motor latency, *CMAP* Compound motor action potential, *CV* conduction velocity, *CMAP* Compound motor action potential, *SNAP* Sensory nerve action potential

To summarize the findings from our case studies and compare them with the treatment outcomes from any previous report, we searched multiple databases, including China National Knowledge Infrastructure (CNKI), Wanfang, Weipu, PubMed, EMBASE, and Cochrane, using the keywords “CIDP”, “psoriasis” and “CIDP complicated with psoriasis”. We identified a few case studies on this topic. For example, in a case report of CIDP with psoriasis, the patient exhibited improvement of sensory disturbances after receiving IVIg treatment at 400 mg/kg/d dose for 5 days. Two weeks later, the patient's psoriasis on the elbows, knees, and hips was also improved significantly, and there was no recurrence-remission during the treatment. This case study suggests that IVIg treatment can not only improve the symptoms of CIDP but also significantly improve psoriasis symptoms and may be an effective treatment for refractory psoriasis [6]. Furthermore, 3 patients with psoriasis reportedly developed CIDP after receiving treatment for anti-tumor necrosis factor alpha (TNF-α). Among them, 2 patients developed CIDP after using adalimumab to treat psoriasis, and 1 patient developed CIDP after using infliximab. But all of them achieved clinical stability after IVIg treatment [7–9] (Table 2).

**Table 2:** Summary of literature on chronic inflammatory demyelinating polyneuropathy (CIDP) in psoriasis patients

Case	Gender	Age (years)	Previous biological treatment	Treatment	The curative effect
1 [6]	male	58	-	One IVIG treatment (400 mg/kg/d for 5 days)	Sensory impairment was significantly improved
2 [7]	male	49	Infliximab (5 mg/kg at weeks 0, 2, and 6, followed by an 8-week interval) was treated for 8 months	Monthly IVIG treatment (400 mg/kg/d, for 5 days)	Limb weakness was significantly improved, and clinical stability was achieved
3 [8]	male	42	Adamuzumab (80 mg twice weekly) was treated for 1 year	IVIG treatment (details not available)	CIDP recovered completely and no recurrence occurred during 5 months of follow-up
4 [9]	female	53	Adamuzumab (40 mg biweekly) was treated for 10 months	Four IVIG treatments (400 mg/kg/d for 5 days); Oral prednisolone (1 mg/kg, reduced at a rate of 10 mg every 2 weeks)	CIDP recovered completely and no recurrence occurred during 12 months of follow-up

Following the worsening of the patient’s recurring symptoms, he was re-admitted to our department, where we integrated the opinions of multidisciplinary experts, such as dermatologists, rheumatologists, and immunologists, to initiate the IL-17A inhibitor, secukinumab, therapy at a dose of 300 mg (1 time/week for 5 weeks; maintenance treatment once every 4 weeks) via subcutaneous injection to control psoriasis symptoms, in combination with IVIg (total dose of 2.0 g/kg, divided into 5 days) and azathioprine(100 mg/day) treatments as the maintenance therapy. Although there is no sufficient evidence to recommend any particular drug as immunosuppressant or immunomodulatory drug, we used azathioprine to reduce the dose and increase the interval of the first-line treatment to find the lowest effective maintenance dose. Later, the patient's symptoms rapidly improved. At the most recent follow-up examinations, the CIDP and psoriasis symptoms are stable.

## Discussion

Presently, the European Society of Neurology/Peripheral Nerve Society (EFNS/PNS) 2021 CIDP guidelines recommend that electrophysiological diagnosis (i.e. nerve conduction examination) play a major role in the precise detection of CIDP, supplemented with neuroimaging, CSF components test, and nerve biopsy [1]. The most effective first-line therapies include corticosteroids, IVIg, and plasma exchange (PE). Corticosteroids have inhibitory effects on a multitude of immune-modulatory functions, such as anti-inflammation, anti-immune, anti-allergic, and anti-shock abilities [10]. Moreover, IVIg can also interfere with the complement activity, production of pro-inflammatory cytokines, and the signal transduction of phagocytes and B cells through Fcγ receptors [11]. Both IA and PE can eliminate pathogenic factors, such as autoantibodies, activated complement, and inflammatory factors, from the blood of patients to achieve rapid and sustained treatment outcomes [12, 13]. In the case of our patient, we found a complete reversal of the first ganglioside autoantibody spectrum results in the subsequent re-examinations when the patient was subjected to the multiple rounds of IVIg and IA treatments, further suggesting that combination of these two approaches may establish potential treatment regimen for CIDP patients complicated with psoriasis.

The patient received IVIg treatment several times. After each IVIg treatment, the symptoms improved, and the MRC sum scores were (0–60) increased by ≥ 6 points. In August 2021, the patient's symptoms recurred with severity to the extent that he was immediately hospitalized. After several IA treatments, the patient's clinical symptoms were significantly relieved, and he was able to walk independently, although a little assistance was required in daily life. Notably, less than half a month later, the patient was re-admitted to the hospital because of severe worsening of the recurring symptoms (Fig. 1). The patient had a history of psoriasis for 6 years in the past. The medications (clobetasol propionate ointment and calcipotriol ointment) were discontinued one year ago, and his psoriasis condition was returned, further worsening the situation. It could be speculated that the CIDP pathogenesis might be related to psoriasis via a common potential immunopathogenic mechanism, which again might be related to the activation of Th17 cells to produce IL-17 and other inflammatory factors (Flowchart 1). Currently, the patient's symptoms have not fluctuated and recurred during the follow-up, and the patient's clinical symptoms have been stabilized for a significantly longer period of time than before (Fig. 1). However, the efficacy of secukinumab still needs to be tested in the long-term follow-up observation. Individuals who are susceptible to autoimmune diseases may develop different kinds of autoimmune diseases, according to the co-stimulatory environment. However, the possibility of accidental onset of the two conditions cannot be ruled out, and further clinical and basic research is needed to confirm it.

## Conclusion

In conclusion, the pathogenesis of CIDP is still unclear and the clinical symptoms are diverse. There are certain difficulties in the selection of treatment options, and the efficacy of CIDP treatment varies greatly between individuals. When CIDP patients do not respond well to the first-line treatment or the course of the disease follows the relapse-remission pattern, it is necessary to consider the possibilities of misdiagnosis, other immune diseases and/or insufficient immunotherapy doses [14]. CIDP combined with psoriasis is clinically a very rare disease phenotype. This patient further reminds clinicians that the clinical manifestations of CIDP are diverse. Therefore, early treatment outcomes in CIDP patients are very crucial, and the close monitoring of the relapse-remission instances along the treatment line can alert the clinicians to the possibility of underlying autoimmune complications, which need to be immediately and efficiently resolved for the long-term stabilization of clinical symptoms in such patients.

## Supplementary Information

**Additional file 1:** **Flowchart 1. **Diagram of the hypothesis of pathogenesis Unknown antigens or environmental factors can trigger the activation of innate immune cells. These activated immune cells can produce a large number of inflammatory factors, such as interleukin (IL)-23 and TNF -α, which can induce the differentiation of primitive T cells into Th17 cells, and the activated Th17 cells then can overproduce IL-17 and IL-22, etc., promoting the proliferation of keratinocytes and recruiting pro-inflammatory cells, such as neutrophils, leading to the development of psoriasis [15]. At the same time, activated Th17 cells and their secreted cytokines IL-17 and IL-22 can destroy the blood-nerve barrier [16], followed by the activation of local intraneural immune responses, macrophage recruitment, and toxic factor secretion leading to myelin sheath injury and the development of CIDP [17]. Il-17 inhibits Schwann cell-mediated myelin regeneration [18]. The combination of factors complicates axonal injury and CIDP, and patients show relapse-remission patterns. Secukinumab can selectively target and bind to IL-17A to inhibit the interaction between IL-17A and Il-17 receptors [19, 20]. Finally, secukinumab can treat psoriasis while achieving clinical stability of CIDP symptoms.

## Data Availability

All datasets generated for this study are included in the article.

## Abbreviations

CIDP: Chronic inflammatory demyelinating polyneuropathy
IVIg: Intravenous injections
MRC: The Medical Research Council
IL: Interleukin
IA: Immunoadsorption
CSF: Cerebrospinal fluid
MRI: Magnetic resonance imaging
TNF-α: Tumor necrosis factor alpha
EFNS/PNS: The European Society of Neurology/Peripheral Nerve Society
PE: Plasma exchange
