# Supplementary material for: Chronic inflammatory demyelinating polyneuropathy and psoriasis comorbidity with significantly alleviated in symptoms after secukinumab: case report
Source: BMC Neurol. 2022 Nov 2;22:400. doi: 10.1186/s12883-022-02928-3 (PMC9628172; doi:10.1186/s12883-022-02928-3)

**Flowchart 1.** Diagram of the hypothesis of pathogenesis

Unknown antigens or environmental factors can trigger the activation of innate immune cells. These activated immune cells can produce a large number of inflammatory factors, such as interleukin (IL)-23 and TNF -α, which can induce the differentiation of primitive T cells into Th17 cells, and the activated Th17 cells then can overproduce IL-17 and IL-22, etc., promoting the proliferation of keratinocytes and recruiting pro-inflammatory cells, such as neutrophils, leading to the development of psoriasis[[15](#_ENREF_15)]. At the same time, activated Th17 cells and their secreted cytokines IL-17 and IL-22 can destroy the blood-nerve barrier[[16](#_ENREF_16)], followed by the activation of local intraneural immune responses, macrophage recruitment, and toxic factor secretion leading to myelin sheath injury and the development of CIDP[[17](#_ENREF_17)]. Il-17 inhibits Schwann cell-mediated myelin regeneration[[18](#_ENREF_18)]. The combination of factors complicates axonal injury and CIDP, and patients show relapse-remission patterns. Secukinumab can selectively target and bind to IL-17A to inhibit the interaction between IL-17A and Il-17 receptors[[19](#_ENREF_19), [20](#_ENREF_20)]. Finally, secukinumab can treat psoriasis while achieving clinical stability of CIDP symptoms.


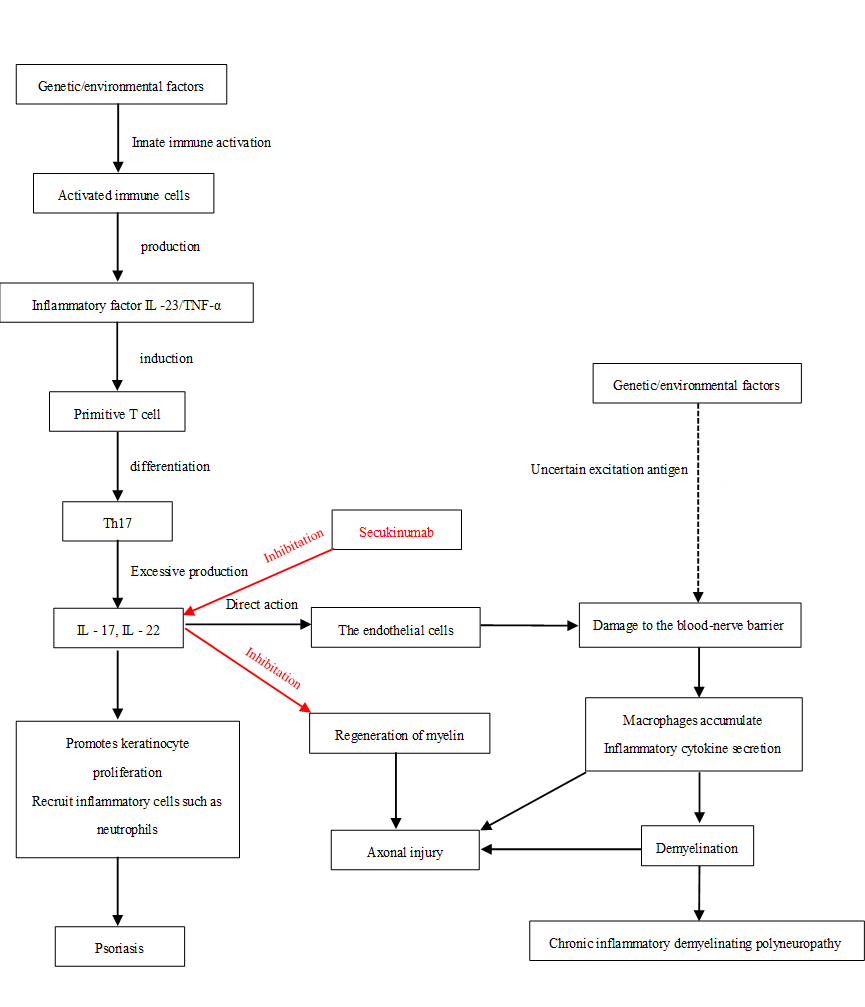

Supplement: Supplementary file 1 — Additional file 1: Flowchart 1. Diagram of the hypothesis of pathogenesis Unknown antigens or environmental factors can trigger the activation of innate immune cells. These activated immune cells can produce a large number of inflammatory factors, such as interleukin (IL)-23 and TNF -α, which can induce the differentiation of primitive T cells into Th17 cells, and the activated Th17 cells then can overproduce IL-17 and IL-22, etc., promoting the proliferation of keratinocytes and recruiting pro-inflammatory cells, such as neutrophils, leading to the development of psoriasis [15]. At the same time, activated Th17 cells and their secreted cytokines IL-17 and IL-22 can destroy the blood-nerve barrier [16], followed by the activation of local intraneural immune responses, macrophage recruitment, and toxic factor secretion leading to myelin sheath injury and the development of CIDP [17]. Il-17 inhibits Schwann cell-mediated myelin regeneration [18]. The combination of factors complicates axonal injury and CIDP, and patients show relapse-remission patterns. Secukinumab can selectively target and bind to IL-17A to inhibit the interaction between IL-17A and Il-17 receptors [19, 20]. Finally, secukinumab can treat psoriasis while achieving clinical stability of CIDP symptoms. [file 12883_2022_2928_MOESM1_ESM.docx]
